# Structure of small HBV surface antigen reveals mechanism of dimer formation

**DOI:** 10.1038/s41421-024-00768-8

**Published:** 2025-01-14

**Authors:** Xiao He, Yunlu Kang, Weiyu Tao, Jiaxuan Xu, Xiaoyu Liu, Lei Chen

**Affiliations:** 1 State Key Laboratory of Membrane Biology, College of Future Technology, Institute of Molecular Medicine, Peking University, Beijing Key Laboratory of Cardiometabolic Molecular Medicine, Beijing, China; 2 Academy for Advanced Interdisciplinary Studies, Peking University, Beijing, China; 3 Peking-Tsinghua Center for Life Sciences, Peking University, Beijing, China; 4 National Biomedical Imaging Center, Peking University, Beijing, China

**Keywords:** Cryoelectron microscopy, Immunology

Dear Editor,

Hepatitis B Virus (HBV) infection can lead to chronic hepatitis B, which significantly increases the risk of death from conditions such as cirrhosis and liver cancer^1^. In 2019, ~296 million individuals were living with chronic hepatitis B, and there were about 1.5 million new infections each year^1^. Moreover, hepatitis B resulted in an estimated 820,000 deaths in 2019, with cirrhosis and hepatocellular carcinoma being the primary causes^1^. HBV is an enveloped virus with HBsAg as the only protein on its envelope. HBsAg exists in three variants: large (L-HBsAg), medium (M-HBsAg), and small (S-HBsAg) surface antigens, all of which contain the amino acids of S-HBsAg. S-HBsAg is predicted to have four helices (H1–H4) with an extracellular N-terminus^2^. The cytosolic loop (CYL) is located between H1 and H2, which is important for HBV virion assembly and maturation^3^. Between H2 and H3, there is a hydrophilic domain known as the “a”-determinant or antigenic loop (AGL), which is essential for the viral infection process^2^. Unlike S-HBsAg, M-HBsAg contains an additional PreS2 region, while L-HBsAg possesses both PreS1 and PreS2 regions. Infected hepatocytes secrete three forms of HBV-related particles: the 42-nm virions, tubular subviral particles (SVPs), and small spherical SVPs with varying sizes^2^. Small spherical SVPs are protein–lipid complexes formed primarily by S-HBsAg dimers and are the major format used in prophylactic HBV vaccines^4^. SVPs do not contain viral genomes and are 10^3^-fold to 10^6^-fold more abundant than virions^2^.

Although the functional significance of HBsAg is well recognized, its structural information only emerges from recent studies. A medium-resolution structure of a small 22-nm spherical SVP at 6.3 Å reveals the overall architecture of the HBsAg dimer and how 24 HBsAg dimers assemble into a rhombicuboctahedral protein complex^5^. Structures of larger 28-nm SVP are resolved at 6.6 Å for D2 symmetry and 8.5 Å for D4 symmetry^6^. Localized reconstruction improved the map of HBsAg dimer to 3.7 Å overall resolution^6^. However, the details of how HBsAg monomers interact to form a dimer at the amino acid level and how the CYL contributes to the structural integrity of the S-HBsAg dimer remain largely unknown. To understand these mechanisms, we sought to determine its structure at high resolution.

Due to limited access to HBV patient serum, we developed a method to recombinantly express the spherical SVP. It is reported that the N-terminus of mature HBsAg is located extracellularly^2^ and appending a signal peptide to L-HBsAg aided high-level production of HBV SVP^7^. Inspired by this, we appended a signal peptide before M-HBsAg constructs (serotype ayw, genotype D3) to direct the insertion of the first transmembrane helix with its N-terminus outside. This approach allowed for the efficient secretion of SVP into the medium of FreeStyle 293F cells. We further purified these SVPs using a strep-tagged scFV fragment of the NAb HBC34 (referred to as NAb_HBC_) (Supplementary Fig. S1a). NAb_HBC_ is the parental antibody of the broad NAb VIR-3434, which is in clinical trials for the treatment of HBV and Hepatitis D Virus (HDV)^8^. NAb_HBC_ recognizes a structural epitope of the AGL of HBsAg^8^. The purified recombinant SVP was subjected to cryo-EM sample preparation and single-particle analysis (Supplementary Figs. S1b, c and S2).

In the raw micrographs and 2D class averages of our cryo-EM sample, we observed only a small 22-nm SVP with no evidence of larger 28-nm SVP or tubular SVP formations (Supplementary Fig. S2a, b). This is different from recent work^6^, in which larger 28-nm SVP dominants were in their sample. We speculate that this may be attributed to differences in expression conditions, such as the presence of an N-terminal flag tag in their study^6^ vs an N-terminal signal peptide in our study. Single-particle reconstruction without symmetry resulted in a map at 4.7 Å resolution with clear helical structural features (Supplementary Fig. S2). The map shows many protrusions from the near-spherical core, representing the AGL regions (Fig. 1a–d). Further inspection revealed that the recombinant SVP has a pseudo-octahedral symmetry. Three HBsAg dimers are arranged around a three-fold symmetry axis to form the HBsAg hexamer (3 × 2 mer) (Fig. 1a). Seven HBsAg hexamers further assemble into the SVP (Fig. 1). This packing also results in additional two-fold (2 × 2 × 2 mer) (Fig. 1b) and four-fold (4 × 2 mer) axes (Fig. 1c).

**Fig. 1:**
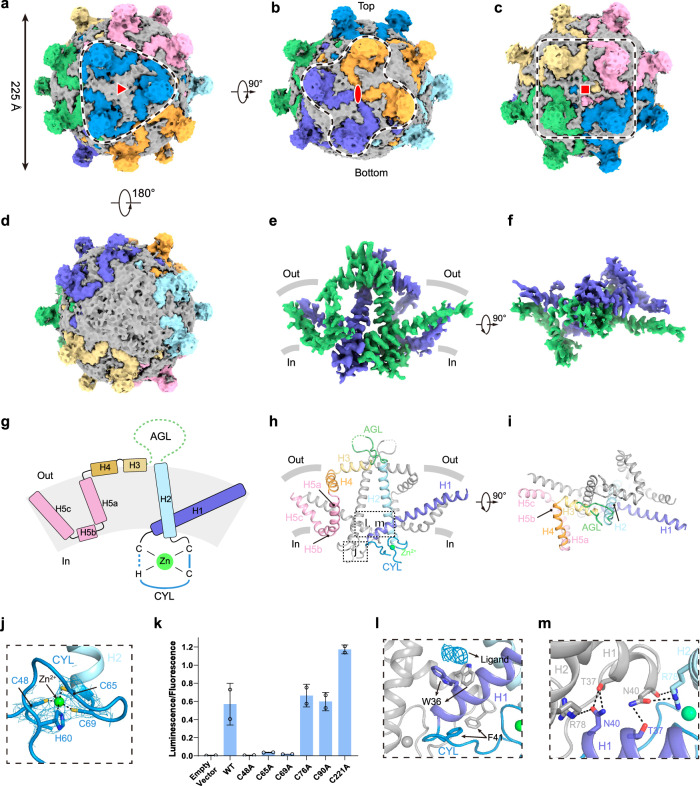
The overall architecture of SVP and structure of HBsAg dimer in SVP. **a** Top view of the cryo-EM map of spherical SVP refined in C1 symmetry. Each HBsAg trimer of dimer (3 × 2 mer) in SVP is colored differently, and the putative lipid density is shown in gray. HBsAg 3 × 2 mer is indicated with a dashed line, and the three-fold axis is indicated in its center. **b** A 90°-rotated view of **a**. HBsAg 2 × 2 × 2 mer is indicated with a dashed line, and the two-fold axis is indicated in its center. Please note the 3 × 2 mer on the top has a strong density, while the density of 3 × 2 mer on the bottom is missing. **c** A 90°-rotated view of **a**. HBsAg 4 × 2 mer is indicated with a dashed line, and the four-fold axis is indicated in its center. **d** A 180°-rotated bottom view of **a**. Please note the density of the HBsAg 3 × 2 mer on the bottom is missing. **e** The side view of the cryo-EM map of HBsAg dimer. Each subunit of the dimer is shown in green (protomer A) or purple (protomer B). The approximate boundaries of the phospholipid bilayer are indicated as thick gray lines. **f** A 90°-rotated top view of **e**. **g** Topology of one of the protomers of HBsAg dimer. Helices, CYL, and AGL are colored differently, and invisible regions in the Cryo-EM map are indicated with dashed lines. **h** Cartoon representation of HBsAg dimer. Helices, CYL, and AGL in protomer A are colored the same as in **g**, and protomer B is shown in gray. **i** A 90°-rotated top view of **h**. **j** Close-up view of CYL boxed in **h**. Structure of CHC2-type zinc finger in CYL. The zinc ion is shown as a bright green sphere, and surrounding cysteines and histidine are shown as sticks. The density of the zinc finger is shown as blue mesh, and the map is contoured at 20 σ. **k** Surface expression of various cysteine mutants of GFP-M-HBsAg. *n* = 2 technical replicates. The experiment was performed three times with similar results. **l** Close-up view of interface area boxed in **h**. Hydrophobic interactions between protomers A and B. Key residues are indicated and shown as sticks. The density of ligand between W36 of two subunits is displayed in blue mesh contoured at 20 σ. **m** Close-up view of interface area boxed in **h**. Hydrophilic interactions between protomers A and B. Key residues are indicated and shown as sticks.

However, the electron density is not uniformly strong across the cryo-EM map, suggesting substantial structural heterogeneity (Fig. 1b). We found that HBsAg 3 × 2 mer at the top of the SVP shows strong density (Fig. 1b), while the density of HBsAg 3 × 2 mer between the top and bottom gradually weakens (Fig. 1b), and the density of HBsAg 3 × 2 mer at the bottom is very weak and hardly visible at low contour levels (Fig. 1b, d), suggesting that the packing of HBsAg hexamers on SVP is heterogeneous and imperfect compared to the standard octahedral symmetry. This phenomenon was not observed previously in 22-nm SVPs purified from patient serum, which showed rhombicuboctahedral symmetry^5^. Whether this discrepancy is due to different expression systems, sample preparation, or single-particle data analysis procedures remains to be elucidated. Because of the deviation from octahedral symmetry in the map (Fig. 1b), forcing reconstruction with octahedral symmetry led to smeared and uninterpretable density maps. Therefore, to improve resolution, we aligned the map to the C3 symmetry axis of the top 3 × 2 mer and subsequent local refinement yielded a consensus map at 4.24 Å resolution (Supplementary Fig. S2c). To improve the resolution, we further subtracted the 3 × 2 mer, 4 × 2 mer, and 2 × 2 × 2 mer from the consensus map and refined them individually by applying local symmetry, resulting in maps at 3.5 Å, 3.9 Å, and 3.1 Å resolution, respectively (Supplementary Fig. S2c). To obtain a better map of the HBsAg dimer, we expanded the HBsAg hexamers (3 × 2 mer) at the top using C3 symmetry and focused the local refinement on a single HBsAg dimer, yielding a map at 3.60 Å averaged resolution (Supplementary Fig. S2c–h). This map was used for model building of the HBsAg dimer core (Supplementary Fig. S3 and Table S1). The architecture of the HBsAg dimer core is overall similar to the previously reported V-shaped structure (Fig. 1e, f)^5,6^, but with enhanced side chain features in many regions (Fig. 1e, f and Supplementary Fig. S3). Although it was predicted that HBsAg contains four helices^2^, we found that residues in the last two predicted helices form three short helices (H3–H5) (Fig. 1g–i). Therefore, we have renamed the helices of HBsAg as H1–H5 according to our current structure (Fig. 1g–i and Supplementary Fig. S4). In addition, H5 is highly curved and is further divided into H5a, H5b, and H5c (Fig. 1g, h and Supplementary Fig. S4). The map quality of the central region of HBsAg is better than other portions (Fig. 1e and Supplementary Fig. S2h), likely reflecting the remarkable structural dynamic of HBsAg. As a result, we observed discrete side chain density for most of H1, CYL, H2, and H3, but the side chain features for H4–H5 are only barely visible for a few bulky residues (Supplementary Fig. S3). Additionally, residues 111–149 of the AGL could not be modeled in this map due to the poor local resolution (Fig. 1e, f), indicating its large conformational heterogeneity.

The structure shows that on the extracellular side, the amphipathic helices H3 and H4 of both HBsAg protomers are located on either side of the AGL and float on the viral envelope (Fig. 1g–i). It is previously reported that the H3 helix is critical for the morphogenesis of SVP^9^, in agreement with its unique position. On the intracellular side, instead of being a flexible loop, the CYL forms a defined structure stabilized by a CHC2-type zinc finger motif, with C48, H60, C65, and C69 coordinating a zinc ion (Fig. 1g, j). To investigate the functional importance of the zinc finger motif, we mutated cysteine residues on the zinc finger or in the transmembrane domains of HBsAg into alanines and detected the surface expression of HBsAg. We found that mutations of cysteines on zinc finger (C48A, C65A, and C69A) abolished the surface expression of HBsAg, while cysteines mutants in the transmembrane domain retain robust surface expression (Fig. 1k), suggesting that the integrity of zinc finger is essential for HBsAg maturation. This observation is consistent with previous findings showing that deleting residues in the CYL impairs virus production^3^ and also correlates with the fact that zinc finger-forming residues are absolutely conserved in HBV-related viruses, while cysteines in the transmembrane domain are not conserved (Supplementary Fig. S4). Interactions between the two HBsAg protomers involve multiple residues at the bottom of H1, CYL, and H2 (Fig. 1l, m). Specifically, W36 on H1 and F41 on CYL interact with their counterparts of the other protomer through hydrophobic interactions (Fig. 1l). We observed a strong ligand density sandwiched between W36 of two subunits, probably representing an unknown hydrophobic molecule. Additionally, R78 on H2 makes polar interactions with the main chain and side chain of N40 on the H1 of the other protomer (Fig. 1m). N40 also makes hydrogen bonding with the T37 of the other protomer. These non-covalent interactions stabilize the HBsAg dimer near the intracellular side, complementing the inter-subunit covalent disulfide linkages in the AGL on the extracellular side. It is important to highlight that the insufficient local map quality in other studies has resulted in the unresolved features of many side chains (Supplementary Fig. S3b, c). Notably, the zinc finger motif of CYL was either not modeled^5^ or incorrectly modeled as a disulfide bond^6^. Detailed structural comparisons reveal that the HBsAg dimer determined in this study is superposable with that obtained from the small 22-nm spherical SVP^5^ (Supplementary Fig. S3d). However, when aligned with the structure derived from the larger 28-nm spherical SVP^6^, only the H1–H3 regions are superposable, while H4–H5 exhibit significant discrepancies (Supplementary Fig. S3e). This suggests that the conformations of H4–H5 of SVP differ due to the distinct packing environments present in the 22-nm and 28-nm SVP structures.

To visualize how HBsAg dimers are assembled into SVP, we docked the HBsAg dimer core structures back into the map of SVP (Supplementary Fig. S5). On the three-fold symmetry axis, the upper regions of H1 from three HBsAg dimers pack together to form a three-helical bundle (Supplementary Fig. S5g). H5c of one dimer interacts with H1 and H2 of adjacent dimers (Supplementary Fig. 5g). Close to the two-fold axis, H5a of one dimer interacts with H5a of the other dimer (Supplementary Fig. S5h). On the four-fold axis, the upper regions of H1 from four dimers pack together to form a four-helical bundle, and H5c of one dimer interacts with H1 of an adjacent dimer (Supplementary Fig. S5i). These inter-dimer interactions stabilize the overall packing and assembly of the 22-nm spherical SVP.

SVP is the major format of the prophylactic HBV vaccines^4^. The high-resolution structures of the HBsAg dimer core and its higher assembly in the small spherical SVP presented here provide a near-atomic resolution understanding of the assembly mechanism of a small 22-nm SVP. This information might offer clues for further drug discovery targeting the folding or maturation of the HBsAg protein or for the structure-based development of next-generation HBV vaccines that are more stable. Furthermore, the highly dynamic nature of H4–H5 helices of HBsAg observed here might be crucial for the plasticity of HBsAg dimer and their assembly into HBV viral envelope or tubular SVP, which might have different packing environments compared to the spherical SVPs.

## Supplementary information

Supplementary material

## Data Availability

Cryo-EM maps and the atomic coordinate of the HBsAg-dimer core have been deposited in the EMDB and PDB under the ID codes EMDB: EMD-61003 and PDB: 9IYX, respectively. Other maps were deposited in the EMDB with codes: EMD-61011, EMD-61012, EMD-61013, EMD-61015, and EMD-61016.
