# Supplementary material for: Structure of small HBV surface antigen reveals mechanism of dimer formation
Source: Cell Discov. 2025 Jan 14;11:6. doi: 10.1038/s41421-024-00768-8 (PMC11730374; doi:10.1038/s41421-024-00768-8)
Supplement: Supplementary file 1 — Supplementary material [file 41421_2024_768_MOESM1_ESM.pdf]

Supplementary Materials for

Structure of small HBV surface antigen reveals mechanism of dimer formation

Xiao He [1], Yunlu Kang [1], Weiyu Tao [1], Jiaxuan Xu [1], Xiaoyu Liu [2],

and Lei Chen [1,2,3,4,*]

[1] State Key Laboratory of Membrane Biology, College of Future Technology, Institute of Molecular Medicine, Peking. University, Beijing Key Laboratory of Cardiometabolic Molecular Medicine, Beijing 100871, China.

[2] Academy for Advanced Interdisciplinary Studies, Peking University, Beijing 100871, China

[3] Peking-Tsinghua Center for Life Sciences, Peking University, Beijing 100871, China

[4] National Biomedical Imaging Center, Peking University, Beijing, 100871, China

*Correspondence: Lei Chen, chenlei2016@pku.edu.cn

This file includes:

Materials and Methods

Additional references

Supplementary Figs. S1 to S5

Supplementary Tables S1

Materials and Methods

Cell Culture

Sf9 insect cells (Thermo Fisher Scientific) were cultured in SIM SF (Sino Biological) at 27 °C. FreeStyle 293F suspension cells (Thermo Fisher Scientific) were cultured in FreeStyle 293 medium (Thermo Fisher Scientific) supplemented with 1% fetal bovine serum (FBS, VisTech), 67 μg ml^{-1} penicillin (Macklin), and 139 μg ml^{-1} streptomycin (Macklin) at 37°C with 6% CO_2 and 70% humidity. The cell lines were routinely checked to be negative for mycoplasma contamination but have not been authenticated.

Constructs

The construct of rSP-M-HBsAg was generated by fusing a rat FSHB signal peptide before the preS2 region of M-HBsAg (serotype ayw, genotype D3). The ORF was cloned into a modified BacMam vector [1]. To generate the construct of $scFv_{HBC}$-strep, the coding sequences of the variable heavy (VH) and light chains (VL) of NAb_{HBC} were synthesized and cloned into a modified BacMam vector, with a GGGGSGGGGSGGGGS-linker between them. A Myc-strep tag was fused to the C-terminal and a rat FSHB signal peptide was fused to the N-terminal. The construct of GFP-M-HBsAg was generated by fusing a GFP with a Strep-Falg-Strep tag on N terminal before the preS2 region of M-HBsAg (serotype ayw, genotype D3), with a PreScission Protease cleavage sequence between them. A rat FSHβ signal peptide sequence was inserted at the N-terminus of the GFP. The ORF was cloned into a modified BacMam vector [1]. Point Mutations on M-HBsAg were introduced using Quikchange PCR.

Expression and purification of small spherical SVP

BacMam virus carrying rSP-M-HBsAg was generated with SF9 cells. 293F cells at a density of 2.5×10^{6} cells mL^{-1} were transfected with 8% volume of P2 virus. 10 mM sodium butyrate was added 12 h after infection and the cells were incubated at 37 °C for 60h before harvest.

After 4000 rpm for 10 min, the cell medium was collected, and the cell debris and insoluble aggregated proteins were removed by 12000 rpm for 15 min (JA 14). To precipitate SVP, 10% PEG6000 (w/v) was added into the collected medium, thoroughly mixed on a magnetic stirrer for 30 min followed by incubation on ice for another 30 min. After 12000 rpm for 15 min (JA 14), the precipitate was collected and resolved with TBS. Insoluble materials were removed by 16000 g for 15 min (JA 14). For purification of SVP, $scFv_{HBC}$-strep was expressed and purified the same as Fab_{HBC}, and the supernatant was added with $scFv_{HBC}$-strep and subjected to strep affinity chromatography to obtain the $scFv_{HBC}$-binding SVPs. The $scFv_{HBC}$-bound SVPs were eluted with buffer containing 50 mM HEPES (pH=8), 150 mM NaCl, and 5 mM desthiobiotin, and subjected to cryo-EM sample preparation.

Cryo-EM sample preparation and data collection

For the SVP sample, the purified $scFv_{HBC}$-SVP was diluted to A_{280} = 2.0. To avoid protein denaturation at the air-water interface, 0.01% glycyrrhizin (estimated concentration of 122 μM) was added to the sample before cryo-EM sample preparation. Holey carbon grids (Quantifoil Au 300 mesh, R 0.6/1) were coated with freshly prepared graphene oxide[2]. Aliquots of 3 μl purified protein were applied on glow-discharged grids and the grids were blotted for 6 s before being plunged into liquid ethane using Vitrobot Mark IV (Thermo Fisher Scientific). Cryo-grids were first screened on a Talos Arctica electron microscope (Thermo Fisher Scientific) operating at 200 kV with a K2 camera (Gatan). The screened grids were subsequently transferred to a Titan Krios electron microscope (Thermo Fisher Scientific) operating at 300 kV with a K3 camera (Gatan) and a GIF Quantum energy filter (Gatan) set to a slit width of 20 eV. Images were automatically collected using EPU (Thermo Fisher Scientific) in super-resolution mode at a nominal magnification of ×81,000, corresponding to a calibrated super-resolution pixel size of 0.533 Å with a preset defocus

range from -1.5 to -1.8 μm. Each image was acquired as a 3.74-s movie stack of 47 frames with a dose rate of 21.46 e^{-} $Å^{-2}$ s^{-1}, resulting in a total dose of about 70 e^{-} $Å^{-2}$.

Cryo-EM image analysis

The image processing workflows are illustrated in Supplementary Figures. Super-resolution movie stacks were collected. Motion-correction, two-fold binning, and dose weighting were performed using MotionCor2 [3]. Contrast transfer function (CTF) parameters were estimated with cryoSPARC [4]. Micrographs with ice or ethane contamination and empty carbon were removed manually. Particles were auto-picked using Gautomatch (provided by K. Zhang). All subsequent classification and reconstruction were performed in cryoSPARC [4] unless otherwise stated. Reference-free 2D classification was performed to remove contaminants. The resulting particles were subjected to 3D classification using the initial models generated from cryoSPARC. Extensive 2D and 3D classification resulted in 530,120 particles, which were subjected to ab initio reconstruction and non-uniform refinement using C1 symmetry, resulting in a map at 4.70Å resolution. The map was aligned to the C3 symmetry axis on the top HBsAg hexamer (3x2mer) and subjected to local refinement using C3 symmetry, resulting in a consensus map at 4.24Å resolution. The 3x2mer on top and 2x2x2mer on side was subtracted out of the particles of this consensus refinement. For 4x2mer, the particles of consensus refinement were expended using C3 symmetry and then used for subtraction. The subtracted particles were subjected to local refinement with corresponding symmetry, yielding maps at 3.49Å for 3x2mer with C3 symmetry, 3.40Å for 4x2mer with C4 symmetry, and 3.90 Å for 2x2x2mer with C2 symmetry. To further improve the resolution of the HBsAg dimer, the particles of locally refined 3x2mer were subjected to symmetry expansion using C3 symmetry and signal subtraction to obtain signals of a single HBsAg dimer. The resulting particles were subjected to local refinement, yielding a map at a resolution of 3.60Å.

Model building

For SVP, the initial model of HBsAg-dimer was predicted with ColabFold [5]. The transmembrane H1 and H2 were fitted into the maps using UCSF Chimera [6] and rebuilt manually in Coot [7]. Model refinement was performed using phenix.real_space_refine in PHENIX [8]. The validation statistics are provided in Tables S1.

Surface labelling of HbsAg

AD293 cells were seeded on 24-well dish coated with poly-D-lysine. After cells were adherent, wild type GFP-M-HBsAg and the mutants were transfected. For surface labeling, the cells were washed with PBS for two times 36 h after transfection, and the whole experiment was performed at room temperature. Cells were incubated with 4 % formaldehyde (10.8% formalin) for 30 min for fixation, and after washed with 500 uL PBS for two times, fixed cells were incubated with 300 uL blocking buffer (3% goat serum in PBS). After 30 min, Hep B preS2 Antibody (Santa Cruz sc-23944, 1:2500 diluted in blocking buffer) was added to specially combine with HBsAg that had been expressed and transported onto cell membrane eventually. After incubation for 1 h, unbonded antibodies were removed by PBS washing for 3 times, and goat anti-mouse IgG conjugated with HRP (Invitrogen 31444, 1:2500 diluted in blocking buffer) was added and incubated for 1 h. After extensive washing with PBS, the cells were added with 250 uL ECL substrate (Tanon 180-5001, 1:20 diluted in water) and reacted for 90 s. Luminescence and fluorescence intensity (488 nm excitation and 520 nm emission) signals were measured with an Infinite M Plex plate reader (Tecan). For normalize, the values of luminescence were divided by GFP fluorescence intensity values.

Quantification and statistical analysis

Global resolution estimations of cryo-EM density maps are based on the 0.143 Fourier Shell Correlation criterion [9]. The local resolution was estimated using cryoSPARC. The number of independent experiments (N) and the relevant statistical parameters for each experiment (such as mean or standard deviation) are described in the figure legends. No statistical methods were used to pre-determine sample sizes.

Additional References

1 Li, N. *et al.* Structure of a Pancreatic ATP-Sensitive Potassium Channel. *Cell* **168**, 101-110 e110, doi:10.1016/j.cell.2016.12.028 (2017).

2 Patel, A., Toso, D., Litvak, A. & Nogales, E. Efficient graphene oxide coating improves cryo-EM sample preparation and data collection from tilted grids. *bioRxiv* (2021).

3 Zheng, S. Q. *et al.* MotionCor2: anisotropic correction of beam-induced motion for improved cryo-electron microscopy. *Nat. Methods* **14**, 331-332, doi:10.1038/nmeth.4193 (2017).

4 Punjani, A., Rubinstein, J. L., Fleet, D. J. & Brubaker, M. A. cryoSPARC: algorithms for rapid unsupervised cryo-EM structure determination. *Nat. Methods* **14**, 290-296, doi:10.1038/nmeth.4169 (2017).

5 Mirdita, M. *et al.* ColabFold: making protein folding accessible to all. *Nat. Methods* **19**, 679-682, doi:10.1038/s41592-022-01488-1 (2022).

6 Pettersen, E. F. *et al.* UCSF Chimera--a visualization system for exploratory research and analysis. *J Comput Chem* **25**, 1605-1612, doi:10.1002/jcc.20084 (2004).

7 Emsley, P., Lohkamp, B., Scott, W. G. & Cowtan, K. Features and development of Coot. *Acta Crystallogr. D Biol. Crystallogr.* **66**, 486-501, doi:10.1107/S0907444910007493 (2010).

8 Afonine, P. V. *et al.* Real-space refinement in PHENIX for cryo-EM and crystallography. *Acta Crystallogr D Struct Biol* **74**, 531-544, doi:10.1107/S2059798318006551 (2018).

9 Chen, S. *et al.* High-resolution noise substitution to measure overfitting and validate resolution in 3D structure determination by single particle electron cryomicroscopy. *Ultramicroscopy* **135**, 24-35, doi:10.1016/j.ultramic.2013.06.004 (2013).

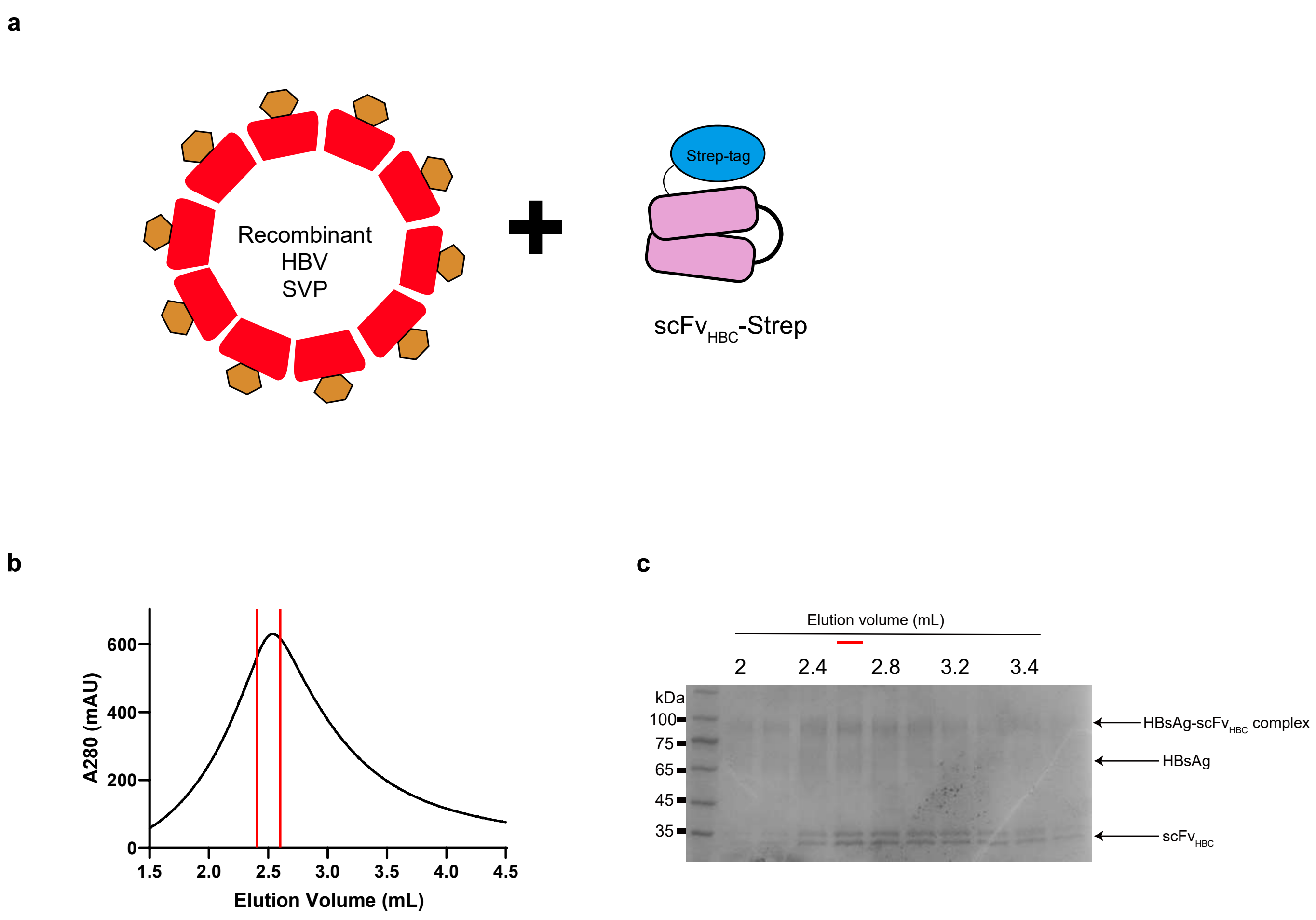

Supplementary Fig. 1 Purification of recombinant small spherical SVP

a, Schematic diagram of the strategy of SVP purification using NAb$_{HBC}$. Affinity chromatography was performed by using the strep tag on the scFv fragment.

b, Strep affinity chromatography of the SVP-scFv$_{HBC}$ complex. The fraction that contains the highest concentration of protein (indicated by red lines) was subjected to cryo-EM sample preparation.

c, Coomassie brilliant blue staining of SDS-PAGE of fractions from affinity chromatography in **b**. The bands corresponding to HBsAg-scFv$_{HBC}$ complex, HBsAg and scFv$_{HBC}$ were indicated. The fractions indicated by the red line were used for cryo-EM sample preparation.

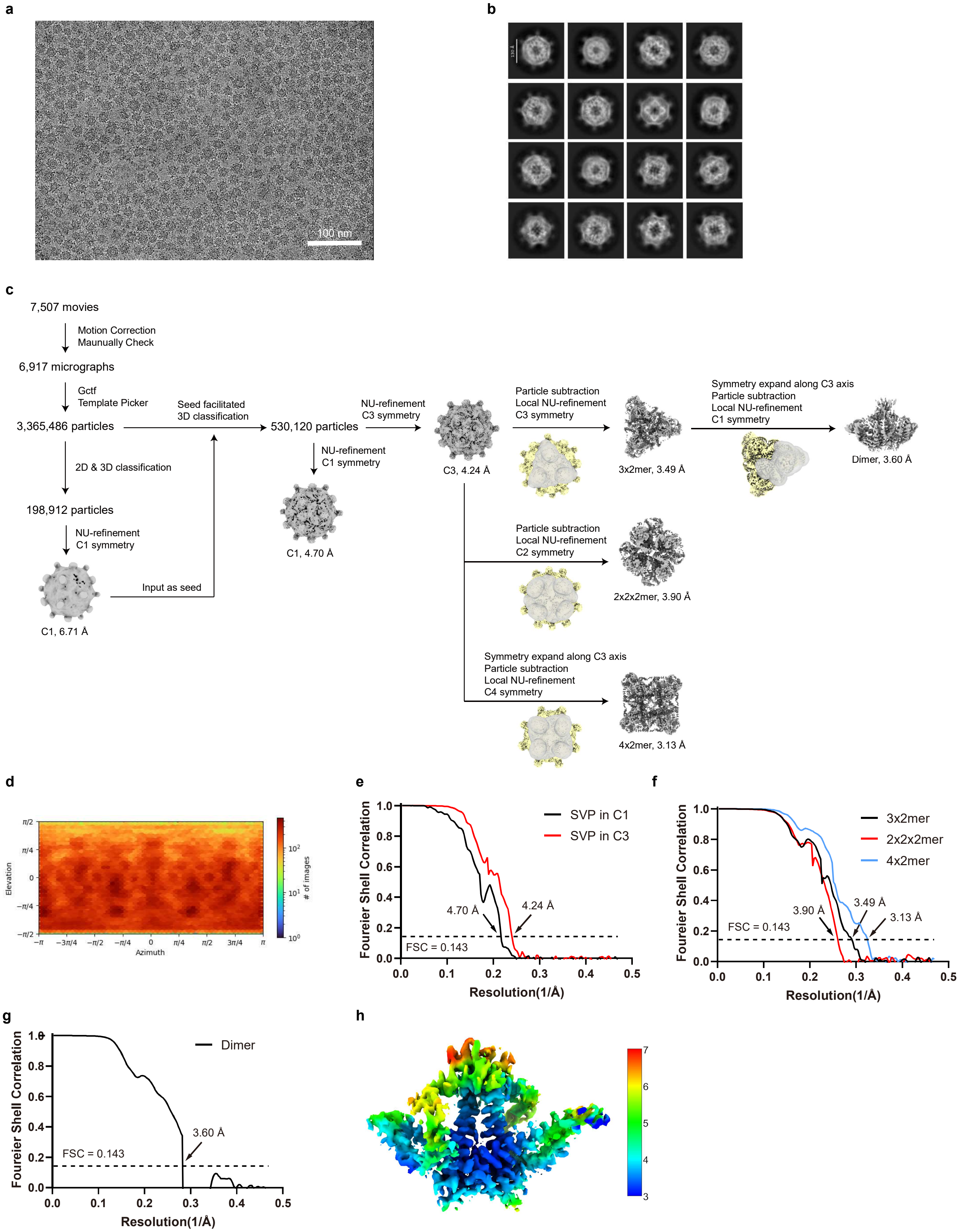

Supplementary Fig. 2 Cryo-EM image analysis of spherical SVP

a, Representative raw micrograph (7,507 in total) of spherical SVP. Scale bar, 100 nm.

b, 2D-class averages of spherical SVP. Scale bar, 130 Å.

c, Cryo-EM data processing workflow of spherical SVP, 3×2mer, 2×2×2mer, 4×2mer and dimer. For details, see 'Cryo-EM image analysis' in the Methods section.

d, Angular distribution of spherical SVP in C1 symmetry.

e, Gold standard Fourier Shell Correlation (FSC) of spherical SVP after correction of masking effects.

f, Gold standard Fourier Shell Correlation (FSC) of HBsAg 3×2mer, 2×2×2mer, and 4×2mer after correction of masking effects.

g, Gold standard Fourier Shell Correlation (FSC) of HBsAg dimer after correction of masking effects.

h, Local resolution map of HBsAg dimer. Scale bar, 3–7 Å.

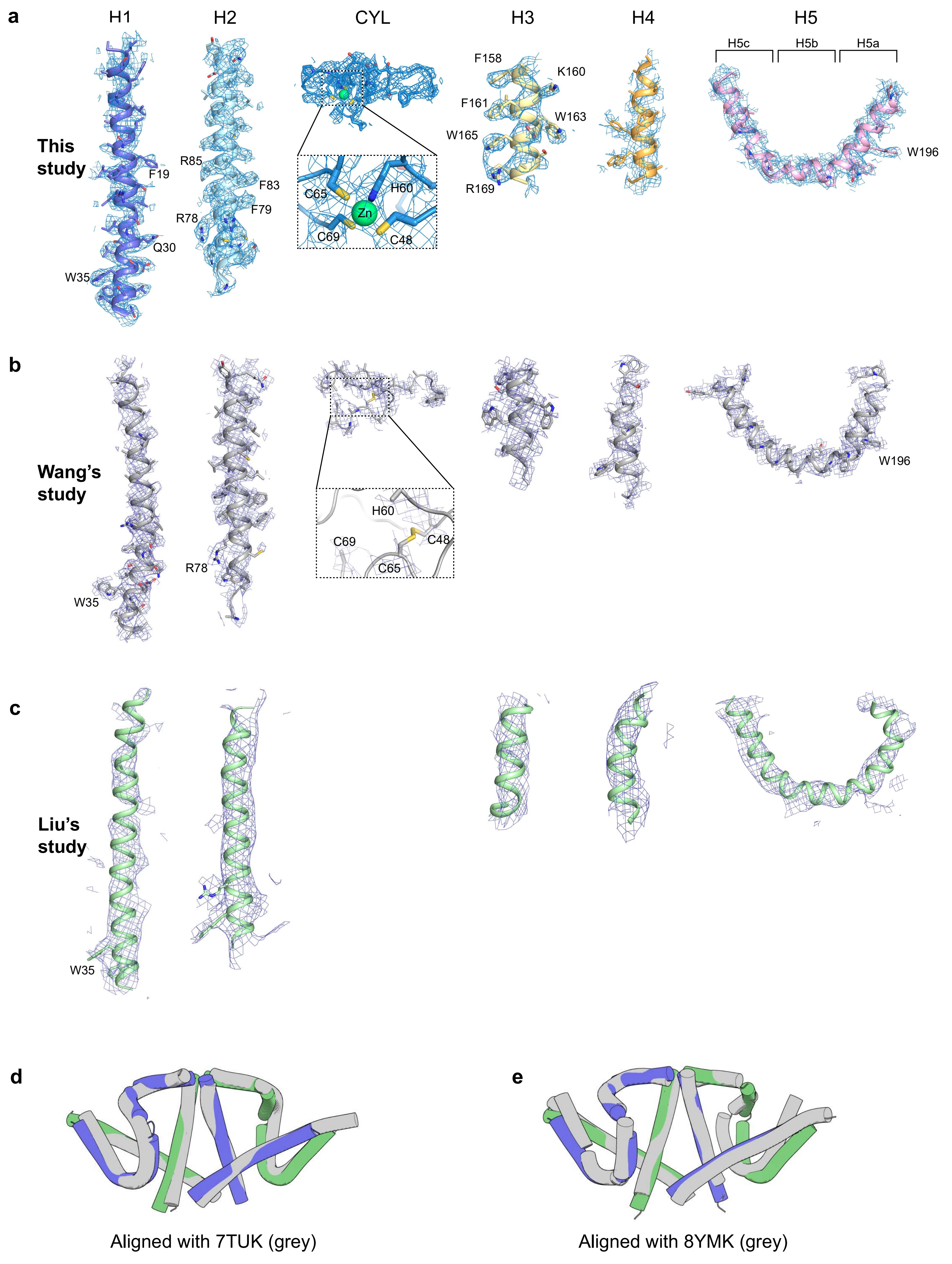

a
H1
H2
CYL
H3
H4
H5
H5c
H5b
H5a
This study
F19
Q30
W35
R85
F83
R78
F79
C65
H60
Zn
C69
C48
F158
K160
F161
W163
W165
R169
W196
b
Wang's study
W35
R78
H60
C69
C48
C65
W196
c
Liu's study
W35
d
Aligned with 7TUK (grey)
e
Aligned with 8YMK (grey)

Supplementary Fig. 3 Representative electron density maps of HBsAg dimer

a, Electron density maps and molecular model obtained in this study. Densities of helices and CYL in HBsAg dimer are shown as blue meshes. The maps are contoured at 20 σ.

b, Electron density maps (emd-39404) and molecular model (PDBID: 8YMK) obtained in Wang's study. Densities of helices and CYL in HBsAg dimer are shown as blue meshes. Incorrect disulfide bond assignment is highlighted. The maps are contoured at 3 σ.

c, Electron density maps (emd-26117) and molecular model (PDBID: 7TUK) obtained in Liu's study. Densities of helices and CYL in HBsAg dimer are shown in blue meshes. The maps are contoured at 3 σ.

d, Structural comparisons of molecular models of HBsAg transmembrane helices, which are shown in cylindrical helices. For the model in this study, protomer A is colored with green, and protomer B is colored with purple, while 7TUK is colored with grey.

e, Structural comparisons of molecular models of HBsAg transmembrane helices, which are shown in cylindrical helices. For the model in this study, protomer A is colored with green, and protomer B is colored with purple, while 8YMK is colored with grey.

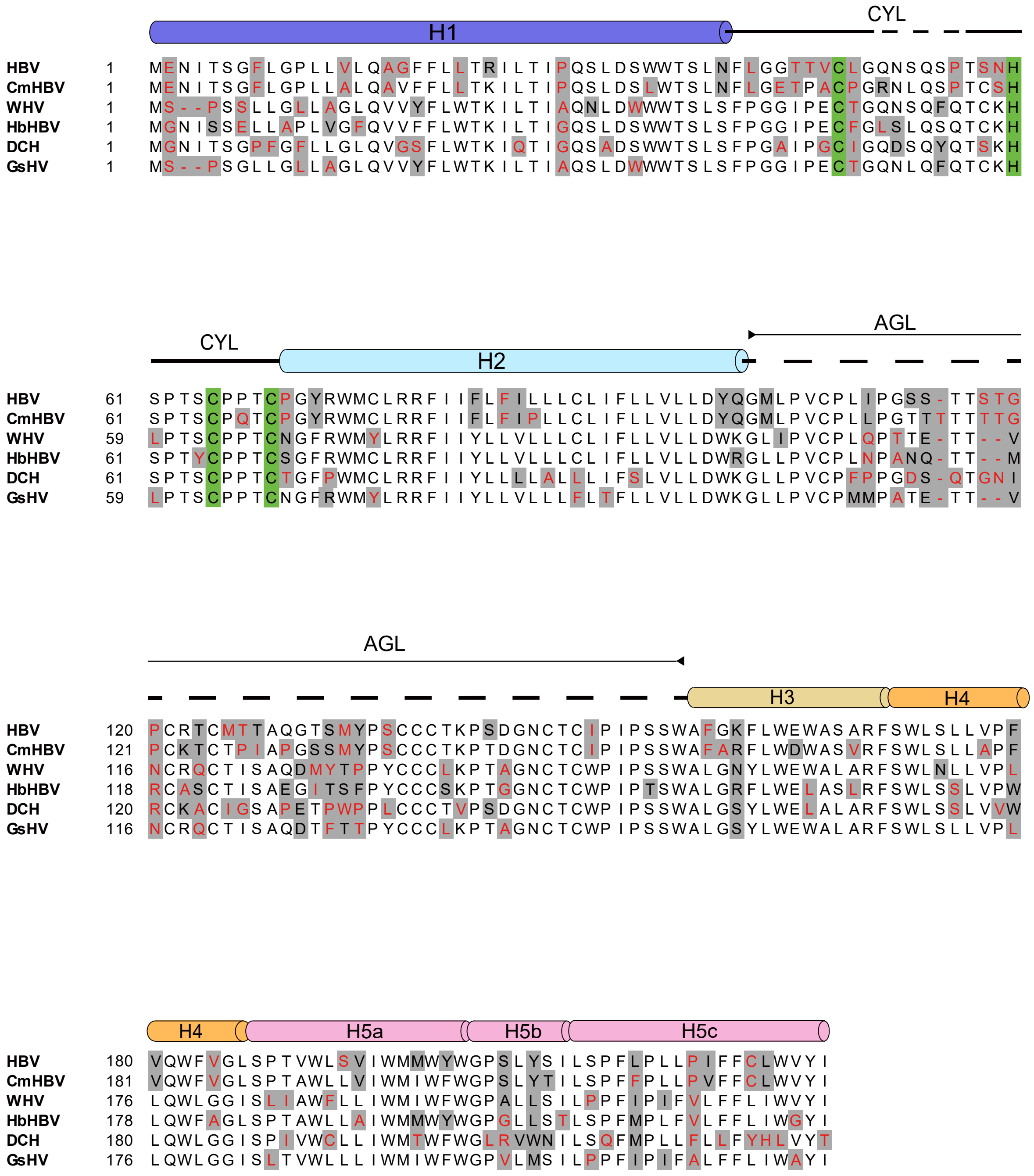

Supplementary Fig. 4 Sequence alignment of the surface antigens of several HBV-related viruses
Multiple sequence alignment (MSA) of S-HBsAg sequences of Hepatitis B virus (HBV, RefSeq: YP_009173871.1), Capuchin monkey hepatitis B virus (CmHBV, RefSeq: YP_009666526.1), Woodchuck hepatitis virus (WHV, RefSeq: NP_944491.1), Horseshoe bat hepatitis B virus (HbHBV, RefSeq: YP_009045996.1), Domestic cat hepadnavirus (DCH, RefSeq: YP_009553237.1) and Ground squirrel hepatitis virus (GsHV, RefSeq: NP_955537.1). The sequences are downloaded from NCBI. Helices are shown as cylinders. Residues which form the intracellular zinc finger motif on CYL are highlighted in green. Similar mutated residues were indicated in grey and the non-conserved residues were highlighted with red letters. Sequence alignment was performed with ClustalW and illustrated by BioEdit.

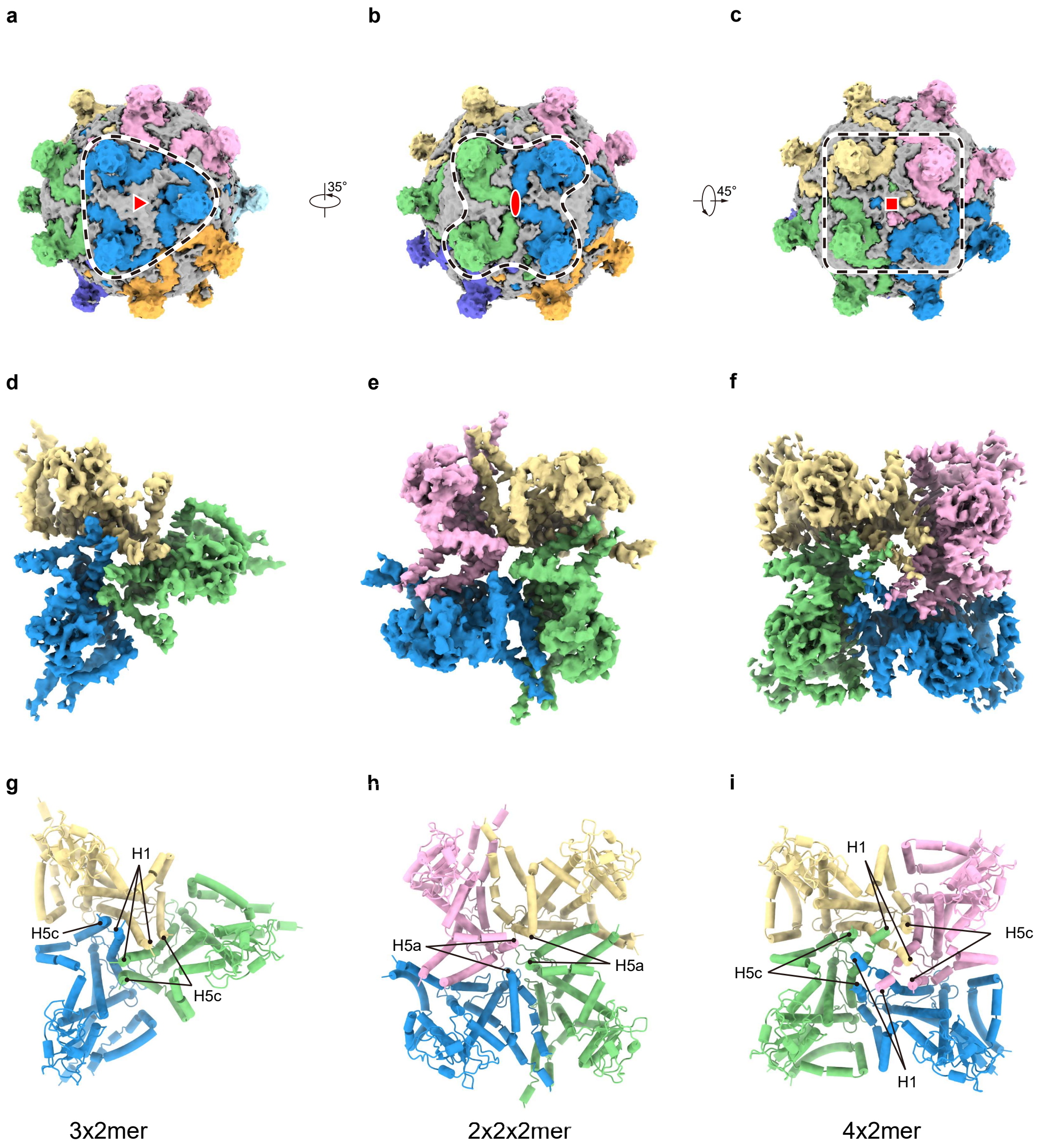

Supplementary Fig. 5 Assembly of HBsAg dimer in spherical SVP

a-c, Different views of the cryo-EM map of spherical SVP refined in C1 symmetry, with HBsAg 3×2mer, 2×2×2mer, and 4×2mer indicated with dashed line, respectively. Each HBsAg trimer of dimer (3×2mer) in SVP is colored differently, and the putative lipid density is shown in grey.

d-f, Cryo-EM map of local refined HBsAg 3×2mer, 2×2×2mer and 4×2mer circled in spherical SVP, respectively.

g-i, Interactions between HBsAg dimer in the assembly of 3×2mer, 2×2×2mer, and 4×2mer respectively. Key helices involved in the assembly are indicated.

Table S1. Cryo-EM data collection, refinement, and validation statistics

The parameters for the Cryo-EM data collection, processing, and validation of HBV SVP are listed in the table.

	SVP in C1 symmetry	SVP in C3 symmetry	HBsAg 3×2mer	HBsAg 2×2×2mer	HBsAg 4×2mer	HBsAg dimer
PDB ID						9IYX
EMDB ID	EMD-61011	EMD-61012	EMD-61013	EMD-61015	EMD-61016	EMD-61003
**Data collection and processing**						
Magnification	81,000 ×					
Voltage (kV)	300					
Electron exposure (e^-/Å^2)	52					
Defocus range (μm)	-1.5 to -1.8					
Pixel size (Å)	1.066					
Symmetry imposed	*C1*	*C3*	*C3*	*C2*	*C4*	*C1*
Initial particle images (no.)	3,365,486					
Final particle images (no.)	530,120	530,120	530,120	530,120	1,560,360	1,560,360
Map resolution (Å)	4.70	4.24	3.49	3.90	3.13	3.60
FSC threshold	0.143	0.143	0.143	0.143	0.143	0.143
Map resolution range (Å)	250-4.70	250-4.24	250-3.49	250-3.90	250-3.13	250-3.60
**Refinement**						
Initial model used (PDB code)						N/A
Model resolution (Å)						3.1
FSC threshold						0.143
Model resolution range (Å)						250-3.1
Map sharpening *B* factor (Å^2)						-139.9
Model composition						
Non-hydrogen atoms						2,626
Protein residues						351
Ligands						2
B factors (Å^2)						
Protein						16.90
Ligand						27.46
R.m.s. deviations						
Bond lengths (Å)						0.005
Bond angles (°)						1.096
Validation						
MolProbity score						1.69
Clashscore						11.06
Poor rotamers (%)						0.40
Ramachandran plot						
Favored (%)						97.35
Allowed (%)						2.65
Disallowed (%)						0.00
